# The genome sequence of the heart cockle, *Fragum sueziense *(Issel, 1869)

**DOI:** 10.12688/wellcomeopenres.22585.1

**Published:** 2024-07-10

**Authors:** Ruiqi Li, Jingchun Li, Sarah Lemer, Jose Victor Lopez, Graeme Oatley, Elizabeth Sinclair, Isabelle Ailish Clayton-Lucey, Eerik Aunin, Noah Gettle, Camilla Santos, Michael Paulini, Haoyu Niu, Victoria McKenna, Rebecca O’Brien, Wellcome Sanger Institute Tree of Life Management, Samples and Laboratory Team, Wellcome Sanger Institute Scientific Operations: Sequencing Operations, Wellcome Sanger Institute Tree of Life Core Informatics Team, EBI Aquatic Symbiosis Genomics Data Portal Team, Aquatic Symbiosis Genomics Project Leadership

**Affiliations:** 1 Ecology & Evolutionary Biology, University of Colorado Boulder, Boulder, Colorado, USA; 2 University of Colorado Boulder Museum of Natural History, Boulder, Colorado, USA; 3 University of Guam Marine Lab, Mangilao, Guam, USA; 4 Leibniz Institute for the Analysis of Biodiversity Change,, Museum of Nature Hamburg, Hamburg, Germany; 5 Department of Biological Sciences, Nova Southeastern University, Dania Beach, Florida, USA; 6 Tree of Life, Wellcome Sanger Institute, Hinxton, England, UK

**Keywords:** Fragum sueziense, heart cockle, genome sequence, chromosomal, Cardiida; Fraginae

## Abstract

We present a genome assembly from an individual *Fragum sueziense* (the heart cockle; Mollusca; Bivalvia; Cardiida; Cardiidae). The genome sequence is 1,206.1 megabases in span. Most of the assembly is scaffolded into 19 chromosomal pseudomolecules. The mitochondrial genome has also been assembled and is 92.77 kilobases in length. Gene annotation of this assembly on Ensembl identified 70,309 protein-coding genes.

## Species taxonomy

Eukaryota; Opisthokonta; Metazoa; Eumetazoa; Bilateria; Protostomia; Spiralia; Lophotrochozoa; Mollusca; Bivalvia; Autobranchia; Heteroconchia; Euheterodonta; Imparidentia; Neoheterodontei; Cardiida; Cardioidea; Cardiidae; Fraginae; *Fragum*; *Fragum sueziense* (Issel, 1869) (NCBI:txid561460).

## Background

The subfamily Fraginae (family: Cardiidae) is renowned for its range of species exhibiting varied ecological adaptations and symbiotic relationships ( Kirkendale, 2009). Among the symbiotic species within the Fraginae subfamily that maintain obligate photosymbiotic relationships with Symbiodiniaceae dinoflagellates, *Fragum sueziense* distinguishes itself through its abundance, broad distribution, and unique ecological adaptations, thriving in deeper habitats compared to other Fragum species ( ter Poorten, 2024). Its distribution spans the Indian and Indo-Pacific Oceans, stretching from the eastern coast of Africa and the Red Sea to the Austral Islands ( ter Poorten, 2024). *F. sueziense* exhibits remarkable habitat diversity, thriving not only in the clean sands of reef environments but also adapting to the murkier conditions of lagoons and large bays ( Kirkendale, 2009). Their relatively smaller size may facilitate adaptation to the reduced light intensity in these deeper habitats, and isotope studies suggest a decreased reliance on photosymbiosis ( Li *et al.*, 2018). Phylogenetic studies indicate that they may represent an earlier divergent lineage within the symbiotic clades of Fraginae ( Li, 2024), and their less modified shell morphology suggests that they could retain ancestral characteristics of symbiotic Fraginae species. The whole genome of *F. sueziense* provides crucial insights into the evolutionary biology and adaptation strategies of the Fraginae subfamily. It can help us understand the genetic underpinnings of its unique ecological niche, particularly its adaptation to deeper, low-light marine environments and reduced reliance on photosymbiosis. Furthermore, the widespread distribution enables us to investigate genetic variability and adaptive differences across various geographic ranges, as well as genetic diversity and connectivity across different populations in marine environments.

## Genome sequence report

**Figure 1.**
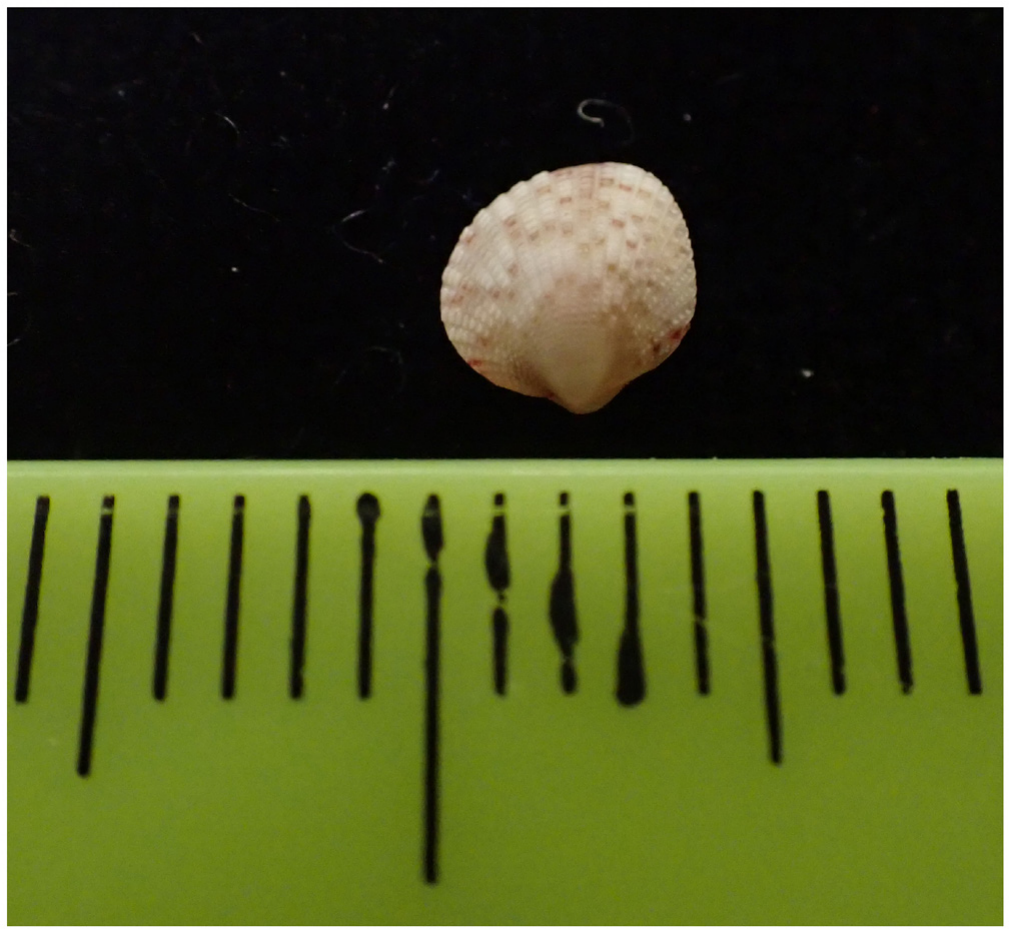
Photograph of the *Fragum sueziense* (xbFraSuez1) specimen used for genome sequencing.

The genome was sequenced from a specimen of *Fragum sueziense* ( Figure 1) collected from Outhouse Beach, Apra Harbor, Guam, USA (13.463, 144.655). A total of 34-fold coverage in Pacific Biosciences single-molecule HiFi long reads was generated. Primary assembly contigs were scaffolded with chromosome conformation Hi-C data. Manual assembly curation corrected 352 missing joins or mis-joins and removed 269 haplotypic duplications, reducing the assembly length by 49.13% and the scaffold number by 29.42%, and decreasing the scaffold N50 by 48.07%.

**Figure 2.**
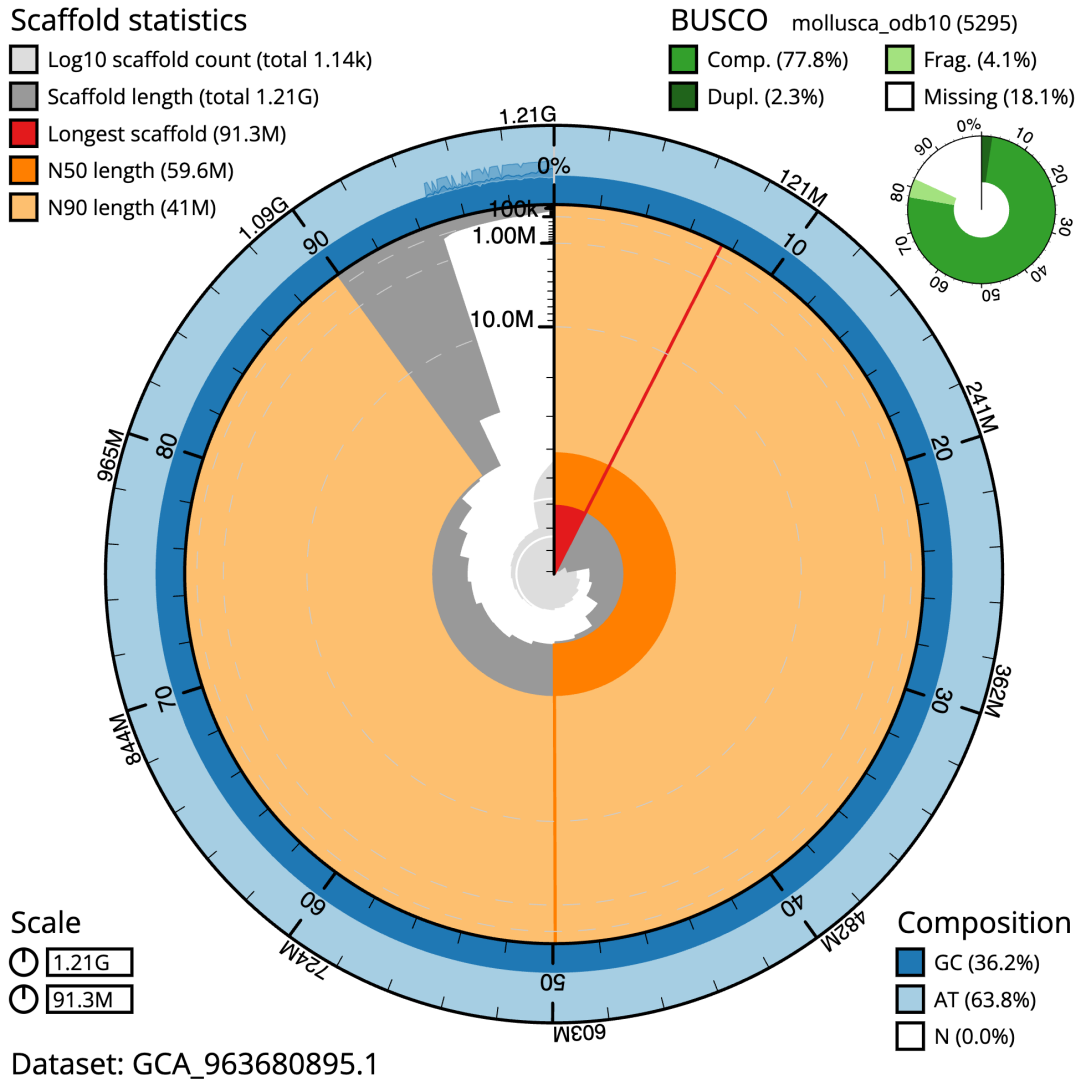
Genome assembly of *Fragum sueziense*, xbFraSuez1.1: metrics. The BlobToolKit snail plot shows N50 metrics and BUSCO gene completeness. The main plot is divided into 1,000 size-ordered bins around the circumference with each bin representing 0.1% of the 1,206,214,559 bp assembly. The distribution of scaffold lengths is shown in dark grey with the plot radius scaled to the longest scaffold present in the assembly (91,300,129 bp, shown in red). Orange and pale-orange arcs show the N50 and N90 scaffold lengths (59,593,990 and 40,995,196 bp), respectively. The pale grey spiral shows the cumulative scaffold count on a log scale with white scale lines showing successive orders of magnitude. The blue and pale-blue area around the outside of the plot shows the distribution of GC, AT and N percentages in the same bins as the inner plot. A summary of complete, fragmented, duplicated and missing BUSCO genes in the mollusca_odb10 set is shown in the top right .An interactive version of this figure is available at https://blobtoolkit.genomehubs.org/view/Fragum_sueziense/dataset/GCA_963680895.1/snail.

**Figure 3.**
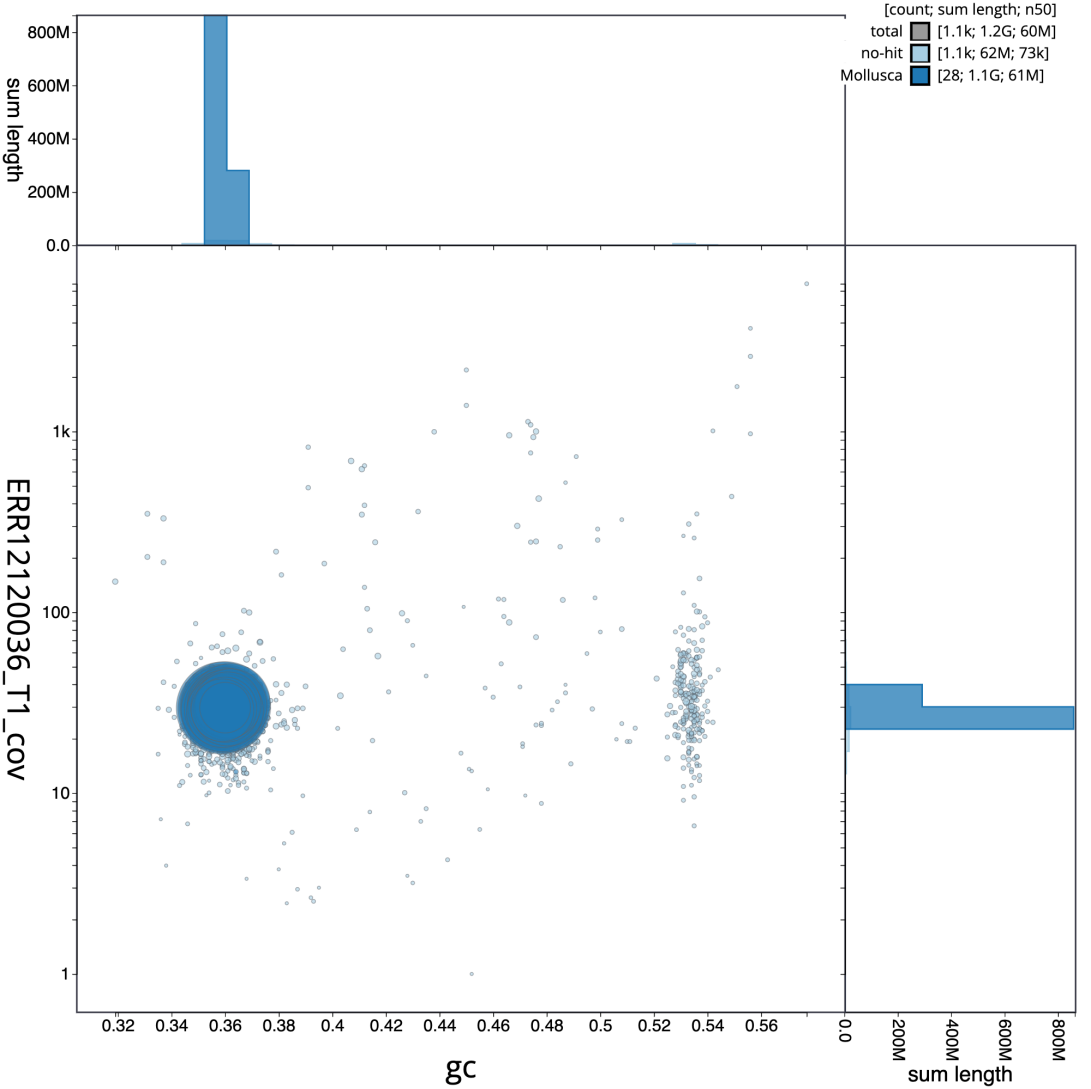
Genome assembly of *Fragum sueziense*, xbFraSuez1.1: BlobToolKit GC-coverage plot. Scaffolds are coloured by phylum. Circles are sized in proportion to scaffold length. Histograms show the distribution of scaffold length sum along each axis. An interactive version of this figure is available at https://blobtoolkit.genomehubs.org/view/Fragum_sueziense/dataset/GCA_963680895.1/blob.

**Figure 4.**
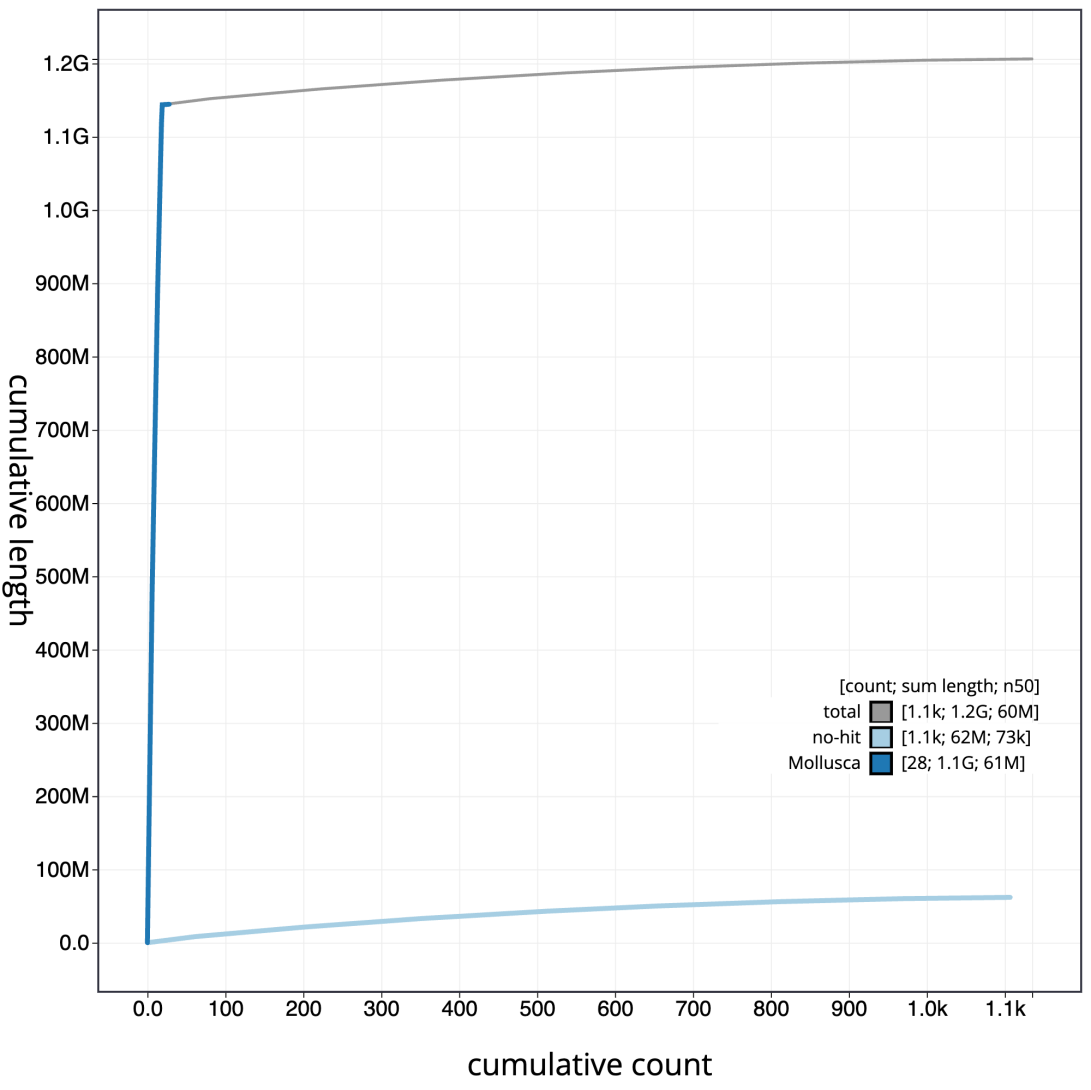
Genome assembly of *Fragum sueziense*, xbFraSuez1.1: BlobToolKit cumulative sequence plot. The grey line shows cumulative length for all scaffolds. Coloured lines show cumulative lengths of scaffolds assigned to each phylum using the buscogenes taxrule. An interactive version of this figure is available at https://blobtoolkit.genomehubs.org/view/Fragum_sueziense/dataset/GCA_963680895.1/cumulative.

**Figure 5.**
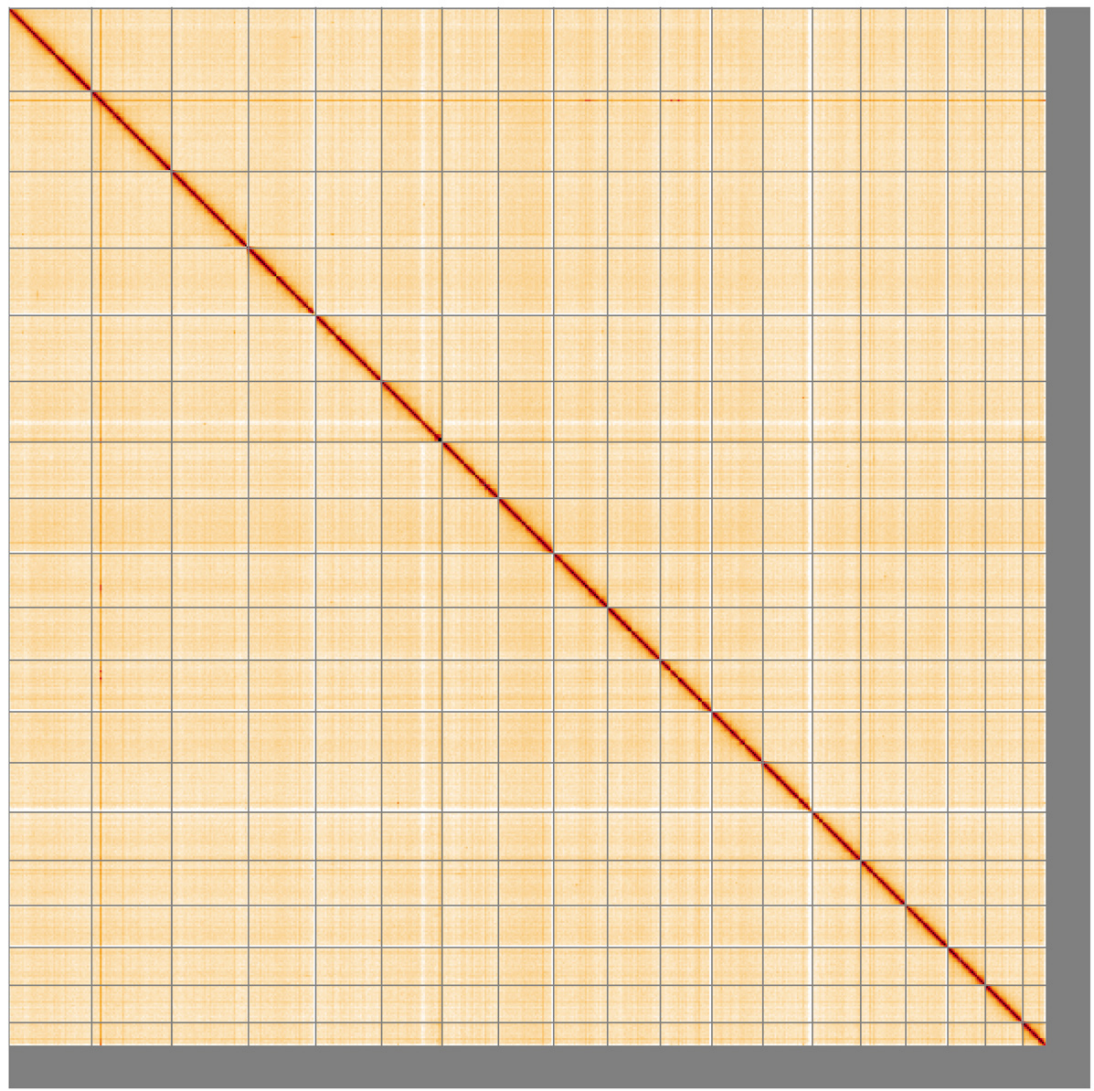
Genome assembly of *Fragum sueziense*, xbFraSuez1.1: Hi-C contact map of the xbFraSuez1.1 assembly, visualised using HiGlass. Chromosomes are shown in order of size from left to right and top to bottom. An interactive version of this figure may be viewed at https://genome-note-higlass.tol.sanger.ac.uk/l/?d=W-A293_JRHOE_RGQ7FQx7A.

The final assembly has a total length of 1,206.1 Mb in 1,134 sequence scaffolds with a scaffold N50 of 59.6 Mb ( Table 1). The snail plot in Figure 2 provides a summary of the assembly statistics, while the distribution of assembly scaffolds on GC proportion and coverage is shown in Figure 3. The cumulative assembly plot in Figure 4 shows curves for subsets of scaffolds assigned to different phyla. Most (94.81%) of the assembly sequence was assigned to 19 chromosomal-level scaffolds. Chromosome-scale scaffolds confirmed by the Hi-C data are named in order of size ( Figure 5; Table 2). While not fully phased, the assembly deposited is of one haplotype. Contigs corresponding to the second haplotype have also been deposited. The mitochondrial genome was also assembled and can be found as a contig within the multifasta file of the genome submission.

**Table 1.** Genome data for *Fragum sueziense*, xbFraSuez1.1.

Project accession data		
Assembly identifier	xbFraSuez1.1	
Species	*Fragum sueziense*	
Specimen	xbFraSuez1	
NCBI taxonomy ID	561460	
BioProject	PRJEB67401	
BioSample ID	SAMEA9449773	
Isolate information	xbFraSuez1	
Assembly metrics *		*Benchmark*
Consensus quality (QV)	60.7	*≥ 50*
*k*-mer completeness	100.0%	*≥ 95%*
BUSCO **	C:77.7%[S:75.3%,D:2.4%], F:4.1%,M:18.2%,n:5,295	*C ≥ 95%*
Percentage of assembly mapped to chromosomes	94.81%	*≥ 95%*
Sex chromosomes	None	*localised homologous pairs*
Organelles	Mitochondrial genome: 92.77 kb	*complete single alleles*
Raw data accessions		
PacificBiosciences SEQUEL II	ERR12120036, ERR12120037	
Hi-C Illumina	ERR12121860	
PolyA RNA-Seq Illumina	ERR12121861	
Genome assembly		
Assembly accession	GCA_963680895.1	
*Accession of alternate haplotype*	GCA_963680815.1	
Span (Mb)	1,206.1	
Number of contigs	3,237	
Contig N50 length (Mb)	0.7	
Number of scaffolds	1,134	
Scaffold N50 length (Mb)	59.6	
Longest scaffold (Mb)	91.3	
Genome annotation		
Number of protein-coding genes	70,309	
Number of non-coding genes	6,440	
Number of gene transcripts	124,524	

* Assembly metric benchmarks are adapted from column VGP-2020 of “Table 1: Proposed standards and metrics for defining genome assembly quality” from Rhie *et al.* (2021). ** BUSCO scores based on the mollusca_odb10 BUSCO set using version 5.3.2. C = complete [S = single copy, D = duplicated], F = fragmented, M = missing, n = number of orthologues in comparison. A full set of BUSCO scores is available at https://blobtoolkit.genomehubs.org/view/Fragum_sueziense/dataset/GCA_963680895.1/busco.

**Table 2.** Chromosomal pseudomolecules in the genome assembly of *Fragum sueziense*, xbFraSuez1.

INSDC accession	Name	Length (Mb)	GC%
OY804592.1	1	91.3	36.0
OY804593.1	2	88.34	36.0
OY804594.1	3	84.54	36.0
OY804595.1	4	74.14	36.0
OY804596.1	5	72.67	36.0
OY804597.1	6	66.73	36.0
OY804598.1	7	61.97	36.0
OY804599.1	8	60.73	36.0
OY804600.1	9	59.59	36.0
OY804601.1	10	58.19	36.0
OY804602.1	11	56.62	36.0
OY804603.1	12	56.46	36.0
OY804604.1	13	54.39	36.0
OY804605.1	14	53.18	36.0
OY804606.1	15	49.72	36.0
OY804607.1	16	46.24	36.0
OY804608.1	17	41.65	36.0
OY804609.1	18	41.0	36.0
OY804610.1	19	26.2	36.0
OY804611.1	MT	0.09	40.5

The estimated Quality Value (QV) of the final assembly is 60.7 with *k*-mer completeness of 100.0%, and the assembly has a BUSCO v5.3.2 completeness of 77.7% (single = 75.3%, duplicated = 2.4%), using the mollusca_odb10 reference set ( *n* = 5,295).

Metadata for specimens, barcode results, spectra estimates, sequencing runs, contaminants and pre-curation assembly statistics are given at https://links.tol.sanger.ac.uk/species/561460.

## Genome annotation report

The *Fragum sueziense* genome assembly (GCA_963680895.1) was annotated at the European Bioinformatics Institute (EBI) on Ensembl Rapid Release. The resulting annotation includes 124,524 transcribed mRNAs from 70,309 protein-coding and 6,440 non-coding genes ( Table 2; https://rapid.ensembl.org/Fragum_sueziense_GCA_963680895.1/Info/Index).

## Methods

### Sample acquisition and nucleic acid extraction

A *Fragum sueziense* (specimen ID NSU0013701, ToLID xbFraSuez1) was collected from Outhouse Beach, Apra Harbor, Guam, USA (latitude 13.463, longitude 144.655) on 2021-06-16 by scuba diving and sifting sand with 1mm metal sifters. The specimen was collected by Sarah Lemer (University of Guam Marine Lab) and identified by Ruiqi Li (University of Colorado Boulder) and preserved by flash freezing in liquid nitrogen.

The workflow for high molecular weight (HMW) DNA extraction at the Wellcome Sanger Institute (WSI) Tree of Life Core Laboratory includes a sequence of core procedures: sample preparation; sample homogenisation, DNA extraction, fragmentation, and clean-up. In sample preparation, the xbFraSuez1 sample was weighed and dissected on dry ice ( Jay *et al.*, 2023). For sample homogenisation, tissue was cryogenically disrupted using the Covaris cryoPREP ^®^ Automated Dry Pulverizer ( Narváez-Gómez *et al.*, 2023). HMW DNA was extracted using the Manual MagAttract v1 protocol ( Strickland *et al.*, 2023b). DNA was sheared into an average fragment size of 12–20 kb in a Megaruptor 3 system with speed setting 30 ( Todorovic *et al.*, 2023). Sheared DNA was purified by solid-phase reversible immobilisation ( Strickland *et al.*, 2023a): in brief, the method employs a 1.8X ratio of AMPure PB beads to sample to eliminate shorter fragments and concentrate the DNA. The concentration of the sheared and purified DNA was assessed using a Nanodrop spectrophotometer and Qubit Fluorometer and Qubit dsDNA High Sensitivity Assay kit. Fragment size distribution was evaluated by running the sample on the FemtoPulse system.

RNA was extracted from tissue of xbFraSuez1 in the Tree of Life Laboratory at the WSI using the RNA Extraction: Automated MagMax™ *mir*Vana protocol ( do Amaral *et al.*, 2023). The RNA concentration was assessed using a Nanodrop spectrophotometer and a Qubit Fluorometer using the Qubit RNA Broad-Range Assay kit. Analysis of the integrity of the RNA was done using the Agilent RNA 6000 Pico Kit and Eukaryotic Total RNA assay.

Protocols developed by the WSI Tree of Life laboratory are publicly available on protocols.io ( Denton *et al.*, 2023).

### Sequencing

Pacific Biosciences HiFi circular consensus DNA sequencing libraries were constructed according to the manufacturers’ instructions. Poly(A) RNA-Seq libraries were constructed using the NEB Ultra II RNA Library Prep kit. DNA and RNA sequencing was performed by the Scientific Operations core at the WSI on Pacific Biosciences SEQUEL II (HiFi) and Illumina NovaSeq 6000 (RNA-Seq) instruments. Hi-C data were also generated from tissue of xbFraSuez1 using the Arima2 kit and sequenced on the Illumina NovaSeq 6000 instrument.

### Genome assembly, curation and evaluation

Assembly was carried out with Hifiasm ( Cheng *et al.*, 2021) and haplotypic duplication was identified and removed with purge_dups ( Guan *et al.*, 2020). The assembly was then scaffolded with Hi-C data ( Rao *et al.*, 2014) using YaHS ( Zhou *et al.*, 2023). The assembly was checked for contamination and corrected using the TreeVal pipeline ( Pointon *et al.*, 2023). Manual curation was performed using JBrowse2 ( Diesh *et al.*, 2023), HiGlass ( Kerpedjiev *et al.*, 2018) and Pretext ( Harry, 2022). The mitochondrial genome was assembled using MitoHiFi ( Uliano-Silva *et al.*, 2023), which runs MitoFinder ( Allio *et al.*, 2020) or MITOS ( Bernt *et al.*, 2013) and uses these annotations to select the final mitochondrial contig and to ensure the general quality of the sequence.

A Hi-C map for the final assembly was produced using bwa-mem2 ( Vasimuddin *et al.*, 2019) in the Cooler file format ( Abdennur & Mirny, 2020). To assess the assembly metrics, the *k*-mer completeness and QV consensus quality values were calculated in Merqury ( Rhie *et al.*, 2020). This work was done using Nextflow ( Di Tommaso *et al.*, 2017) DSL2 pipelines “sanger-tol/readmapping” ( Surana *et al.*, 2023a) and “sanger-tol/genomenote” ( Surana *et al.*, 2023b). The genome was analysed within the BlobToolKit environment ( Challis *et al.*, 2020) and BUSCO scores ( Manni *et al.*, 2021; Simão *et al.*, 2015) were calculated.

Table 3 contains a list of relevant software tool versions and sources.

**Table 3.** Software tools: versions and sources

Software tool	Version	Source
BlobToolKit	4.2.1	https://github.com/blobtoolkit/blobtoolkit
BUSCO	5.3.2	https://gitlab.com/ezlab/busco
Hifiasm	0.16.1-r375	https://github.com/chhylp123/hifiasm
HiGlass	1.11.6	https://github.com/higlass/higlass
Merqury	MerquryFK	https://github.com/thegenemyers/MERQURY.FK
MitoHiFi	2	https://github.com/marcelauliano/MitoHiFi
PretextView	0.2	https://github.com/wtsi-hpag/PretextView
purge_dups	1.2.3	https://github.com/dfguan/purge_dups
sanger-tol/genomenote	v1.0	https://github.com/sanger-tol/genomenote
sanger-tol/readmapping	1.1.0	https://github.com/sanger-tol/readmapping/tree/1.1.0
YaHS	1.1a.2	https://github.com/c-zhou/yahs

### Genome annotation

The Ensembl gene annotation system ( Aken *et al.*, 2016) was used to generate annotation for the *Fragum sueziense* assembly (GCA_963680895.1). Annotation was created primarily through alignment of transcriptomic data to the genome, with gap filling via protein-to-genome alignments of a select set of proteins from UniProt ( UniProt Consortium, 2019).

### Wellcome Sanger Institute – Legal and Governance

The materials that have contributed to this genome note have been supplied by a Tree of Life collaborator. The Wellcome Sanger Institute employs a process whereby due diligence is carried out proportionate to the nature of the materials themselves, and the circumstances under which they have been/are to be collected and provided for use. The purpose of this is to address and mitigate any potential legal and/or ethical implications of receipt and use of the materials as part of the research project, and to ensure that in doing so we align with best practice wherever possible. The overarching areas of consideration are:

• Ethical review of provenance and sourcing of the material

• Legality of collection, transfer and use (national and international)

Each transfer of samples is undertaken according to a Research Collaboration Agreement or Material Transfer Agreement entered into by the Tree of Life collaborator, Genome Research Limited (operating as the Wellcome Sanger Institute) and in some circumstances other Tree of Life collaborators.

## Data Availability

European Nucleotide Archive: *Fragum sueziense* (heart cockle). Accession number PRJEB67401; https://identifiers.org/ena.embl/PRJEB67401 ( Wellcome Sanger Institute, 2023). The genome sequence is released openly for reuse. The *Fragum sueziense* genome sequencing initiative is part of the Aquatics Symbiosis Genomics (ASG) project. All raw sequence data and the assembly have been deposited in INSDC databases. Raw data and assembly accession identifiers are reported in Table 1.

## Author information

Members of the Wellcome Sanger Institute Tree of Life Management, Samples and Laboratory Team are listed here: https://doi.org/10.5281/zenodo.10066175.

Members of the Wellcome Sanger Institute Scientific Operations: Sequencing Operations are listed here: https://doi.org/10.5281/zenodo.10043364.

Members of the Wellcome Sanger Institute Tree of Life Core Informatics team are listed here: https://doi.org/10.5281/zenodo.10066637.

Members of the European Bioinformatics Institute ASG Data Portal team are listed here: https://doi.org//10.5281/zenodo.10076466.

Members of the Wellcome Sanger Institute/Aquatic Symbiosis Genomics Project Leadership are listed here: https://doi.org/10.5281/zenodo.10184833.
