# Supplementary material for: The genome sequence of the heart cockle, Fragum sueziense (Issel, 1869)
Source: Wellcome Open Res. 2024 Jul 10;9:366. [Version 1] doi: 10.12688/wellcomeopenres.22585.1 (PMC11467648; doi:10.12688/wellcomeopenres.22585.1)
Supplement: Peer review history

# Reviewer report: Reviewer response for version 1

**DOI:** 10.21956/wellcomeopenres.24883.r100690

**Published:** 2024-10-21

**Authors:** Qinggang Xue, Sheng Liu

**Affiliations:** 1 Zhejiang Wanli University, Ningbo, Zhejiang, China

The article presents a heart cockle genome assembly from a *Fragum sueziense* individual *. *This work was rationalized by the unique ecological attributes of the analyzed species, as the genomic information generated can be used for the investigation of some important ecological problems. I, however, have two questions:

  1. The numbers of generated contig and scaffold, as shown in Table 1 and Fig 3, were unusually higher as compared to most known bivalve mollusk genome assemblies. Were these differences noticed and addressed by, for example, using alternative assemblage methods?

 2. Also unusual was the number of predicted/annotated protein coding genes, which was 2-3 fold of that the most reported bivalve genomes. Was this technologically addressed in the work?

Are sufficient details of methods and materials provided to allow replication by others?

Yes

Is the rationale for creating the dataset(s) clearly described?

Yes

Are the datasets clearly presented in a useable and accessible format?

Yes

Are the protocols appropriate and is the work technically sound?

Yes

Reviewer Expertise:

bivalve functional genomics

We confirm that we have read this submission and believe that we have an appropriate level of expertise to confirm that it is of an acceptable scientific standard.

# Reviewer report: Reviewer response for version 1

**DOI:** 10.21956/wellcomeopenres.24883.r100697

**Published:** 2024-10-15

**Authors:** Kyle McElroy

**Affiliations:** 1 Department of Ecology, Evolutionary, and Organismal Biology, Iowa State University, Ames, Iowa, USA

The authors report details on the assembly and annotation of a chromosome-level genome assembly for the heart cockle, *Fragum sueziense*. The assembly appears to be high quality and should be useful resource for studying – as the introduction describes – adaptation to deep-water habitats. I think the methods are clear and the rationale behind sequencing this species are presented well. I have a few minor comments and one (somewhat) bigger concern about the mitochondrial genome assembly.

**Major concern**

 I’m curious about the quality of the mitochondrial (mt) genome assembly. Do the MitoHiFi output include evidence of a single circular molecule? Given the rather extreme size of this mt genome, the full annotation (i.e., genes identified with MitoHiFi) should be reported, too. I also think showing the coverage based on the PacBio HiFi and Illumina reads would be helpful. Again, the 90Kb size is notable – it’s among the largest ever reported for a bilaterian animal – and some more attention to this result is warranted. The genome assemblies for *Fragum whitleyi *and *Fragum fragum* on GenBank have 30Kb and 22Kb long mt assemblies, so even compared to congeners the *F. sueziense* mt genome is large.

 The number of protein coding genes from the nuclear genome is also quite high. I think more detail on the annotation process is needed to assess this result. For example, were repeats identified and masked before gene prediction?

**Minor comments**

 I think a brief description of the Hi-C protocol in *Sample acquisition and nucleic acid extraction* would be useful. Right now, there’s only a mention of the kit used in *Sequencing*.

 I would report on the Metazoa BUSCO scores, too. I’ve found that even high-quality assemblies can have poor scores from the Mollusca database.

 In the *Genome annotation report* section, the number of transcripts and genes is stated, and Table 2 is referenced. I believe this is the second table in the annotation page on Ensembl but could be confused with Table 2 in this report, which only includes stats for the pseudomolecules (accession, length, GC%). Perhaps cite this result as “Gene counts table available from: https://rapid.ensembl.org/Fragum_sueziense_GCA_963680895.1/Info/Index”.

Are sufficient details of methods and materials provided to allow replication by others?

Partly

Is the rationale for creating the dataset(s) clearly described?

Yes

Are the datasets clearly presented in a useable and accessible format?

Yes

Are the protocols appropriate and is the work technically sound?

Yes

Reviewer Expertise:

Evolutionary biology, genomics, genome evolution, bivalves, gene-family evolution, opsins, asexual reproduction, transposable elements

I confirm that I have read this submission and believe that I have an appropriate level of expertise to confirm that it is of an acceptable scientific standard, however I have significant reservations, as outlined above.

# Reviewer report: Reviewer response for version 1

**DOI:** 10.21956/wellcomeopenres.24883.r100691

**Published:** 2024-10-10

**Authors:** Jessica Goodheart

**Affiliations:** 1 Division of Invertebrate Zoology, American Museum of Natural History, Scripps Institution of Oceanography, University of California San Diego, New York, La Jolla, USA

This publication outlines a new genome for the heart cockle, *Fragum sueziense*. The methods in this manuscript appear to be clear and sufficient, and  the reasoning for sequencing and publication are sound. I have a couple of small points:

 One thing that is not clear to me is whether this is a symbiotic species.

 The low BUSCO score I assume is due to the use of the Mollusca BUSCO database, so it would be helpful to know how this genome fares with regard to the metazoan or eukaryote databases.

 The BlobToolKit GC-coverage plot has a distinct cluster of no-hit sequences to the right of the primary Mollusca cluster. It is unclear if this is likely due to contamination or some other factor.

Are sufficient details of methods and materials provided to allow replication by others?

Yes

Is the rationale for creating the dataset(s) clearly described?

Yes

Are the datasets clearly presented in a useable and accessible format?

Yes

Are the protocols appropriate and is the work technically sound?

Yes

Reviewer Expertise:

Molluscan genomics, phylogenetics, and evolution

I confirm that I have read this submission and believe that I have an appropriate level of expertise to confirm that it is of an acceptable scientific standard.

# Reviewer report: Reviewer response for version 1

**DOI:** 10.21956/wellcomeopenres.24883.r92500

**Published:** 2024-08-27

**Authors:** Paola Venier, Enrico Bortoletto

**Affiliations:** 1 University of Padova, Padova, Italy; 2 Department of Biology, University of Padova, Italy, Padova, Italy

I introduce myself and Enrico Bortoletto as co-reviewers.

 The open access article under review illustrates a chromosome-scale genome assembly obtained from one individual heart cockle *Fragam sueziense *( *Cardiidae* family). This concise Data Note applies the workflow and procedures in use for genome projects at the Wellcome Sanger Institute and makes available an accurate report of the genome assembly, well supported by hyperlinked data and publicly available protocols. The results generated by the authors will push forward new investigations on heart cockles.

 Describing this small bivalve, it would be worth mentioning Li R. et al., 2024 ( *F. sueziense* extracellularly hosts photoautotrophic microeukaryotes from the genus *Cladocopium*, Symbiodiniaceae family).

 Here below, we add some comments aimed to understand the robustness of the newly generated data.

 The suboptimal BUSCO score C: 77.7% obtained with BUSCO 5.3.2 against orthologous genes from Mollusca (Tables 1 and 3) would deserve some interpretative hints to make clear what biological/technical factors must be critically considered in similar studies. Could the metazoan_odb10, instead of mollusca_odb10, provide a better score? Besides, the authors may consider BUSCO v. 5.7.1 and Compleasm ( https://github.com/huangnengCSU/compleasm ; Huang and Li, 2023).

 Given that the gene content of mtDNA is around 37 genes, with sizes of 16-18 kb in common bivalve species, the length of mtDNA of *F. sueziense* seems excessive (92.77 kb) also when compared to that of *Ruditapes philippinarum* (22 kb; NC_031332), *Anadara broughtonii *(48.6 kb; OM807134), and *Dreissena polymorpha* (67.2 kb; CM035931). See “Bivalvia mitochondrion complete genome” within the NCBI nucleotide sequences. It is not clear whether the mt DNA data of *F. sueziense* excludes or suggests a Double Uniparental Inheritance or DUI (Doucet-Beaupré et al., 2010; Ghiselli et al., 2011; Parakatselaki and Ladoukakis 2021; Kartavtsev and Masalkova 2024).

 As regards the main figures, the assembly statistics reported in the snailplot (Fig. 2) are not easily readable. In the GC-coverage plot (Fig. 3) a minimal explanation should be given to the small light blue dots, scaffolds of minimal length not attributable to *F. sueziensis*. As minor note, the cumulative sequence plot (Fig. 4) is not interactive as said in the legend.

 Incidentally, the 36% GC content uniformly reported for each pseudochromosome seems an average value and it is not clear is *F. sueziensis* displays AT/GC heterogeneity at the scaffold level (Vohnoutová et al., 2023).

Are sufficient details of methods and materials provided to allow replication by others?

Yes

Is the rationale for creating the dataset(s) clearly described?

Yes

Are the datasets clearly presented in a useable and accessible format?

Yes

Are the protocols appropriate and is the work technically sound?

Yes

Reviewer Expertise:

genetics and genetic toxicology, functional genomics, microbiology

We confirm that we have read this submission and believe that we have an appropriate level of expertise to confirm that it is of an acceptable scientific standard.

## References

[1] : Comparative transcriptomics revealed parallel evolution and innovation of photosymbiosis molecular mechanisms in a marine bivalve. *Proc Biol Sci* .2024;291(2023) : 10.1098/rspb.2023.2408 20232408 10.1098/rspb.2023.2408 38807516 PMC11285757

[2] : compleasm: a faster and more accurate reimplementation of BUSCO. *Bioinformatics* .2023;39(10) : 10.1093/bioinformatics/btad595 10.1093/bioinformatics/btad595 37758247 PMC10558035

[3] : Mitochondrial phylogenomics of the Bivalvia (Mollusca): searching for the origin and mitogenomic correlates of doubly uniparental inheritance of mtDNA. *BMC Evol Biol* .2010;10: 10.1186/1471-2148-10-50 50 10.1186/1471-2148-10-50 20167078 PMC2834691

[4] : Strict sex-specific mtDNA segregation in the germ line of the DUI species Venerupis philippinarum (Bivalvia: Veneridae). *Mol Biol Evol* .2011;28(2) : 10.1093/molbev/msq271 949-61 10.1093/molbev/msq271 20952499

[5] : mtDNA Heteroplasmy: Origin, Detection, Significance, and Evolutionary Consequences. *Life (Basel)* .2021;11(7) : 10.3390/life11070633 10.3390/life11070633 34209862 PMC8307225

[6] : Structure, Evolution, and Mitochondrial Genome Analysis of Mussel Species (Bivalvia, Mytilidae). *Int J Mol Sci* .2024;25(13) : 10.3390/ijms25136902 10.3390/ijms25136902 39000014 PMC11241113

[7] : Abandoning the Isochore Theory Can Help Explain Genome Compositional Organization in Fish. *Int J Mol Sci* .2023;24(17) : 10.3390/ijms241713167 10.3390/ijms241713167 37685974 PMC10487504
